# Healthcare utilization and hospital variation in cardiac surveillance during breast cancer treatment: a nationwide prospective study in 5000 Dutch breast cancer patients

**DOI:** 10.1186/s40959-020-00068-6

**Published:** 2020-08-08

**Authors:** Yvonne Koop, Saloua El Messaoudi, Hester Vermeulen, Angela H. E. M. Maas, Femke Atsma

**Affiliations:** 1 Department of Cardiology, Radboud University Medical Center, Geert Grooteplein 10 - route 616, Postbus 9101, 6500 Nijmegen, HB The Netherlands; 2 Scientific Institute for Quality of Healthcare, Radboud University Medical Center, Nijmegen, The Netherlands; 3 Research Department of Emergency and Critical Care, Faculty of Health and Social Studies, HAN University of Applied Sciences, Nijmegen, The Netherlands

**Keywords:** Cardiac imaging, Epidemiology, Breast cancer, Cardiotoxicity, Quality of care, Health policy

## Abstract

**Background:**

Various breast cancer (BC) treatments, such as chemotherapy and targeted therapies, increase cardiotoxicity-risk and lead to premature ischemic heart disease and heart failure among survivors. Reducing this adverse risk through early recognition and (preventive) treatment is therefore important. Conversely, we feel that screening for cardiotoxicity is currently insufficiently standardized in daily practice. A fundamental first step in identifying areas of improvement is providing an overview of current practice.

**Objective:**

This study aims to describe current cardiac surveillance for women with BC during and after cardiotoxic cancer treatment, using routinely collected hospital data in the Netherlands. The study also describes hospital variation in cardiac surveillance.

**Methods:**

This observational study was performed on claims data provided by Statistics Netherlands. From the data, newly diagnosed BC patients in 2013 (*N* = 16,040) were selected and followed up until 2015. Healthcare utilization analyses were performed for all cardiac and oncologic healthcare activities but with a specific focus on cardiac surveillance healthcare activities. In addition, differences between types and individual hospitals were evaluated.

**Results:**

Almost one third of all BC patients received high risk cardiotoxic treatments (*N* = 5157), but cardiac surveillance was rarely performed. Cardiac care provided to patients mainly consisted of ECGs (52.0%) and MUGA scans (26.5%). Cardiac MRI was performed in 0.7% of the patients, echocardiography in 17.7%, and measurement of Troponin and NT-proBNP in 5.1 and 5.8%, respectively. Moreover, we observed a substantial variation in cardiac surveillance between different hospital types and between individual hospitals.

**Conclusion:**

This study shows that women treated for BC with cardiotoxic treatments do not receive recommended cardiac surveillance. Standardized approaches in clinical care are lacking, resulting in low rates of diagnostic testing and a substantial variation in surveillance between hospitals. A structured approach and increased interprofessional collaboration could lead to tailored cardiac surveillance for early detection of cardiotoxicity and therefore start of treatment.

## Introduction

The number of breast cancer (BC) survivors has increased rapidly due to impressive achievements in BC screening and treatment [1]. Anthracycline and trastuzumab are the cornerstone of current BC regimens, and their cardiotoxic effects are well established [2, 3]. The most common clinical presentation of anthracycline-induced cardiotoxicity is a dose-dependent cardiomyopathy, leading to heart failure with a reduced survival. Receiving additional trastuzumab in women with HER-2-positive BC increases this risk even more [2].

Chemotherapy-induced cardiomyopathy was traditionally considered to have a poor prognosis and was often refractory to heart failure treatment. Recent studies, however, suggest that reduction in left ventricular ejection fraction (LVEF) can be mitigated when cardiotoxicity is detected at an early stage and when there is timely medical treatment [4, 5]. Risk stratification, followed by early detection, monitoring, and treatment of cardiotoxicity in high-risk BC patients (i.e. cardiac surveillance), is essential to further improve prognosis. In particular, patients without a cardiovascular (CV) history who have not been monitored or previously assessed by a cardiologist may be overlooked. Preventive strategies focus on markers for detecting early myocardial damage that can predict the development of subsequent reductions in LVEF [6]. With novel imaging techniques, such as strain echocardiography, and cardiovascular magnetic resonance (CMR), signs of myocardial damage can be detected as early as 5 weeks after chemotherapy [7]. Moreover, cardiac biomarkers, such as Troponin and NT-proBNP, can be useful in the detection of cardiotoxicity [6].

In current clinical practice, protocols based on current recommendations are not yet present; therefore, the extent to which cardiac surveillance is performed in BC patients is largely unknown. In this study, we describe the healthcare utilization (HCU) of Dutch women who have been newly diagnosed with BC and who have a high medication-related cardiotoxicity-risk despite not having any CV history. We also examine the hospital variation in cardiac surveillance provided to BC patients in the Netherlands.

## Methods

### Data source

We performed an observational study to report the HCU of BC patients receiving cardiotoxic cancer treatment. Our analysis included nationwide hospital claims data from 2013 up to and including 2015—the most recent data available at the time of this study. Data was provided by Statistics Netherlands (CBS, The Netherlands). Hospital claims data consist of Diagnosis-Related-Groups that comprise actual healthcare activities. These are registered for all insured citizens, covering over 99% of the Dutch population [8]. We had data available on age, sex, and medical history. Healthcare activities included outpatient visits, inpatient admissions, and the performance of imaging modalities, laboratory tests and oncologic treatments. We also obtained available data on type of hospital where the patients received their treatment (university medical centers and non-academic hospitals). In the Netherlands, non-academic hospitals are divided in top clinical hospitals, general hospitals and private hospitals. Top-clinical hospitals deliver both basic and complex care, they are often specialized in delivering care to one or more specific populations. General hospitals provide basic care and private hospitals are mainly specialized in a specific type of care. Information about behavioral CV risk factors, such as smoking or sedentary behavior, are not available in the claims data.

### Study population

We included female patients aged 18 and above with newly diagnosed malignant neoplasm of the breast (ICD-10 codes C50.0-C50.9) in 2013 in the Netherlands [8]. All patients with any cardiac or oncologic diagnosis from 1 January 2012 up to their BC diagnosis in 2013 were excluded because we are specifically interested in a population with high cardiotoxicity-risk based on cardiotoxic cancer treatment and without any history of cardiovascular disease (CVD) or prior cancer treatment. We defined BC patients with high CV risk according to their cardiotoxicity-risk score [9]. High medication-related risk included treatment with anthracyclines, alkylating agents (e.g. cyclophosphamide, ifosfamide), antimetabolite (e.g. clofarabine), and trastuzumab (Table 1).

**Table 1:** Clusters of healthcare activities

Surgery	Surgeries registered under breast cancer (BC) diagnosis codes (specified in study population), for instance lumpectomy, mastectomy and lymph node removal
Chemotherapy	Standard chemotherapy, such as anthracyclines, alkylating agents and anti-metabolites
Targeted therapy	Targeted therapies, such as trastuzumab and pertuzumab
Radiotherapy	Radiation and radiotherapy fractions.
Hormone therapy	Selective estrogen receptor modulators (SERM) and aromatase inhibitors (AI)
Laboratory test	All lab tests regarding hematology and chemistry
Imaging	All conventional radiology (ultrasounds, doppler, duplex), MRI scans, SPECT, PET and CT
Nuclear imaging	SPECT, PET and MUGA
Diagnostic tests (other than imaging)	Non-invasive and invasive diagnostics, such as, electrocardiography, diagnostic puncture, colonoscopy
Multi-disciplinary consultations	Co-treatments of other specialists, (clinical) multidisciplinary consultations and activities
Emergency care	Contact with emergency department, life support.
Hospital admissions	A first or subsequent clinical admission,
Outpatient visits	A first or return visit
Tele-consultations	A first or return consultation by phone

*MRI* Magnetic Resonance Imaging, *CT* Computed Tomography, *SPECT* Single-photon emission computed tomography, *PET* positron emission tomography

Standard dosage in Dutch clinical protocols for doxorubicin is 60 mg/m2. Each cycle is always combined with cyclophosphamide 600 mg/m2 for which patients usually receive 4 cycles. For trastuzumab, a loading dose of 8 mg/kg is given, followed by a standard dosage of 6 mg/kg. In addition, women with BC who did not receive any of these high-risk cardiotoxic treatments were selected for comparison. Patients either received lower-risk (mono) chemotherapy (e.g. paclitaxel), radiotherapy, hormone therapy, or surgery.

### Healthcare utilization categories

From the time of their inclusion when diagnosed with primary BC in 2013, patients were monitored until the end of 2015, resulting in follow-up periods varying from 2 to 3 years for individual patients. We included both baseline cardiac monitoring prior to cancer treatment initiation and after the start of cancer treatment. To describe HCU, we clustered healthcare activities within categories of care. We first drafted the following HCU categories related to both BC and CVD: laboratory tests, imaging, other diagnostic tests, treatments, inpatient admissions, and outpatient visits. The categories were then discussed, clarified, and refined in two meetings: (1) with experts in the research team (i.e. cardiologist, oncologist, epidemiologist, clinical health scientist) and (2) with four cardiology experts (i.e. cardiologists and cardiology researchers). Throughout the data interpretation and analysis, oncologists were consulted. This approach resulted in 14 definite HCU categories, as presented in Table 1. These categories encompass diagnostic and prognostic tests (e.g. imaging, laboratory tests) related to BC and CVD as well as hospital visits (e.g. outpatient visit, emergency care). Healthcare activities coded as ‘other’ or those without a specific description were excluded from the clustering process (< 4%) along with rare healthcare activities (*n* ≤ 5). In the end, 83% of all healthcare activities were clustered in the predefined categories.

### Data analysis

We used the HCU clusters to provide an overview of cardiac surveillance provided to patients. Proportions of HCU represent the percentage of patients who received a specified type of care at least once during the follow-up period. We then analyzed the degree in variation of provided cardiac surveillance between different types of hospitals (e.g. university and non-academic hospitals (i.e. top-clinical hospital, general hospital)) and between individual hospitals. Private hospitals were excluded because they rarely treat BC patients (*N* ≤ 30) with specific oncologic therapies; hence, they are not representative of the standard of care. The proportion of provided care was calculated by dividing the number of patients receiving a specific type of cardiac care at least once from a specific hospital by the number of patients treated for BC in that specific hospital. To account for small sample variations, we only included hospitals with more than 30 newly diagnosed BC patients. We used R for statistical computing and graphics (R Development Core Team, 2008, Vienna, Austria) to conduct all statistical analysis. The Strengthening the Reporting of Observational Studies in Epidemiology (STROBE) guideline was used to enhance reporting quality and transparency.

### Ethics

The Medical Research Ethics Committee of Arnhem-Nijmegen provided a waiver since the study does not require an ethical review. The study was conducted in accordance with the principles of ICH Good Clinical Practice, applicable privacy requirements, and guiding principles of the Declaration of Helsinki.

## Results

### Patient population

In 2013, 16,040 female patients received their first diagnosis of BC. These patients had a mean age of 65.9 (± 12.9), and most of them (90.6%) were born in the Netherlands. The incidence rates per BC stage in the Netherlands in 2014 were as follows: 53.2% early stage BC (DCIS, stage I), 42.2% advanced breast cancer (stages II, IIIa, IIIb/c), and 4.6% metastatic disease (stage IV) [10]. In our final analysis, we included only high-risk patients who received high-risk cardiotoxic treatments consisting of chemotherapy or targeted therapy and those with no cardiac or oncologic history at the moment of BC diagnosis (*N* = 5157). Included patients had a mean age of 59.3 (± 10.5). Of these patients, 98.6% received chemotherapy, while 26.9% received targeted therapy. Patients often received multimodality treatment: 25.5% received both chemotherapy and targeted therapy, 58.9% also received hormone therapy, and 67% were treated with additional radiotherapy. Based on therapy received, 26.9% had HER2+ status, while 58.9% had ER+ and/or PR+ status.

### Overall healthcare utilization

Included patients received their care from 94 different hospitals: 8 university hospitals, 31 top-clinical hospitals, and 55 general hospitals. Most patients were treated in general and/or top-clinical hospitals. Table 2 presents the HCU of BC patients treated with cardiotoxic regiments. In this population, all patients had at least one outpatient visit during follow-up; all outpatient visits in the follow-up period (i.e. 2–3 years) were included. Incidence of inpatient admission (98.6%) and presentation at the emergency department (49%) appeared high; all indications for admission, including BC-related surgery admission, were included in these proportions.

**Table 2:** Overall and oncological healthcare utilization in breast cancer patients in 2013–2015

Healthcare type	***N*** = 5157	***N*** = 10,883
	BC patients with high cardiotoxicity risk (%) ^**a**^	BC patients with lower cardiotoxicity risk (%) ^**a**^
Activities performed by		
Cardiologists	36.6	22.9
HCU related to BC treatment		
Diagnostic tests	87.9	62.5
Imaging	96.6	92.6
Treatment		
Chemotherapy	98.6	10.0
Targeted therapy	26.9	0
Hormone therapy	58.9	21.7
Radiotherapy	67.0	40.6
Surgery	86.2	57.4
Overall healthcare utilization		
Multi-disciplinary consultations	51.5	34.6
Emergency care visit	49.0	19.1
Hospital admission day	98.6	64.9
Outpatient visit	100	99.4
Tele-consultations	84.7	48.7

*HCU* Healthcare Utilization, ^a^Each specific type of care is depicted with the percentage of patients for whom this type of care was registered at least once

### Cardiac surveillance

Among included patients, 36.6% received a type of cardiac care at least once during follow-up (see Table 3). Cardiac care provided consisted mostly of the performance of an ECG (52%) and/or a MUGA scan (26.5%). An echocardiogram was performed in 17.7% of the patients, whereas 0.7% had a CMR. Coronary calcium scores (cardiac CT) were measured in 0.4% of patients. Cardiac biomarkers, such as Troponin I or T and/or NT-proBNP were determined in respectively 5.1 and 5.8% of the patients. Similarly, an exercise stress test was performed in 3.7% of the patients and a 24-h Holter monitoring in 2.4%.

**Table 3:** Cardiologic healthcare utilization in breast cancer patients in 2013–2015

Healthcare activities	***N*** = 5157	***N*** = 10,883
	BC patients with high cardiotoxicity risk (%) ^**a**^	BC patients with lower cardiotoxicity risk (%) ^**a**^
Laboratory tests		
Troponin I/T	5.1	3.8
BNP/ NT-proBNP	5.8	2.9
Imaging		
CMR	0.7	0.2
Echocardiography	17.7	8.7
CT with coronary calcium score	0.4	0.2
Cardiac nuclear imaging	27.7	1.2
MUGA scan	26.5	0.4
Diagnostic tests		
ECG	52.0	26.5
Exercise stress test	3.7	4.1
24-h Holter monitoring	2.4	2.1
Angiography	0.4	0.5
With FFR	0.1	0.1
With IVUS	0.02	0
Reveal	0.02	0.06

^a^Each specific type of care is depicted with the percentage of patients for whom this type of care was registered at least once. *BNP* B-type Natriuretic Peptide, *NT-proBNP* N-terminal pro b-type Natriuretic Peptide, *CMR* Cardiovascular Magnetic Resonance, *CT* Computed Tomography, *MUGA* Multigated acquisition, *ECG* Electrocardiography, *FFR* Fractional Flow Reserve, *IVUS* Intravascular Ultrasound

### Variation in cardiac surveillance between hospitals

Of the 94 hospitals, we excluded 6 general hospitals because of low numbers (< 30 patients) or because they do not deliver any type of cardiac care for their BC patients, leaving 88 hospitals for the hospital variation analysis.

**Fig. 1:**
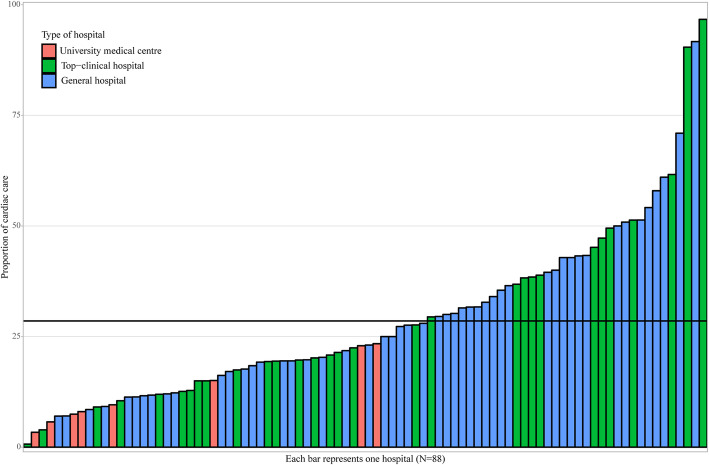
Proportion of cardiac care in breast cancer patients with cardiotoxic treatment. Specified for hospital types and individual hospitals

We observed a substantial variation between individual hospitals and between different types of hospitals. Between individual hospitals, the proportion of cardiac surveillance varied from 0.7 to 96.7%. In general hospitals, this proportion ranged from 7.0 to 91.7%, with a mean of 30.3%. In top-clinical hospitals, the mean proportion of cardiac surveillance was 28.6% (range 0.7–96.7%), while in university hospitals, this was 12.2% (range 3.4–23.4%). The variations in cardiac surveillance between hospitals delivering BC care are described in Table 4 and Fig. 1.

**Table 4:** Concentration of cardiologic healthcare utilization in breast cancer patients in 2013–2015

BC patients with high cardiotoxicity risk (***N*** = 5157)					
Type of hospital	Number of hospitals^**a**^ (N)	Mean age of treated BC patients (years)	Proportion cardiac surveillance^**b**^ (%)	Proportion range (%)	Proportion 5/95 percentile (%)
University hospital	8	53.8	12.2	3.4–23.4	4.2–23.2
Top-clinical hospital	30	54.7	28.6	0.7–96.7	6.2–77.5
General hospital	50	56.2	30.3	7.0–91.7	8.8–59.7

^a^6 hospitals were excluded based on small samples (< 30 patients treated) and not delivering cardiac surveillance, ^b^Proportion of patients treated for BC receiving cardiac surveillance in a hospital. Calculated with the number of patients treated for BC in a hospital and of these patients the number receiving cardiac surveillance

### Comparison group

In addition to the high-risk group, we analyzed HCU of a group of patients without high-risk cardiotoxic BC treatment. Overall, these patients had fewer BC treatments than high-risk patients, with 10% receiving chemotherapy, 40.6% receiving radiotherapy, and 21.7% receiving hormone therapy (see Table 2). Cardiac surveillance for the lower-risk population was largely comparable to the high-risk population (see Table 3) although the proportion of imaging was lower. HCU was comparable to laboratory tests, CMR, and CT with coronary calcium score as well as angiography, stress test, and Holter monitoring. Proportions of echocardiography (8.7%), MUGA (0.4%), and ECG (26.5%) were lower in the comparison group.

## Discussion

The present study is the first to provide an overview of cardiac surveillance for BC patients in current clinical practice. Although almost one third of all BC patients received cardiotoxic treatments, cardiac surveillance was rarely performed. In addition, we observed substantial variation between hospitals. In current clinical practice, there is no observed difference between cardiac surveillance proportions of high and low cardiotoxicity-risk patients. Apart from imaging, both high- and low-risk patients received largely comparable surveillance.

The results of the present study indicate that cardiac surveillance with imaging and laboratory tests during and after cancer treatment are not performed routinely, which is in line with other studies reporting low cardiac surveillance proportions and supports the conclusion that more attention and understanding are needed to deliver recommended cardiac surveillance for this population [11–13]. In this study, cardiac monitoring consisted mainly of the performance of an ECG or a MUGA scan, which in clinical practice are often routinely performed before BC treatment. ECGs, however, are not the most relevant diagnostic tests to detect cardiotoxicity. Cardiac surveillance with imaging (e.g. *echo*, MRI, or MUGA) at baseline and every 3 months is highly recommended for patients receiving trastuzumab and/or anthracycline therapy [14]. Dutch clinical protocols often recommend MUGA for HER2+ BC patients receiving trastuzumab but not for those receiving anthracyclines. Chavez et al. (2015) studied adherence to these recommendations in patients receiving trastuzumab-based therapy and found an adherence rate of only 36% [12].

Additionally, monitoring with MUGA is not the most optimal imaging technique for the detection of early myocardial damage. We found, however, that this imaging modality was most often performed. With MUGA scans, patients are repeatedly exposed to radiation, and MUGA has a low positive and negative predictive value for the detection of cardiotoxicity resulting in incorrect cardiotoxicity diagnosis (in means of underdiagnosis) in 35% of the cases [15]. With MUGA the only measured parameter is LVEF, however subclinical myocardial deformation (as a sign of cardiotoxicity) occurs prior to any LVEF changes [16]. Thus, for early detection of cardiotoxicity MUGA has limited value. More accurate imaging techniques (e.g. *echo*cardiography and CMR) can detect cardiac damage at an earlier stage, and this can have important therapeutic consequences in treating or preventing cardiac damage [17, 18]. Changes in global longitudinal strain (GLS) can be detected prior to LVEF reduction. A relative reduction of 11% compared to baseline or a value of < 19% is an indication of cardiotoxicity [17, 19]. Additionally, novel CMR multi-parametric techniques, such as T1 and T2 mapping, allow accurate characterization of myocardial tissue. With these techniques, early signs of chemotherapy-induced cardiomyopathy, such as myocardial edema and diffuse fibrosis, can be detected [20, 21].

Hospital variation in cardiac surveillance in BC was substantial. The overall proportion of cardiac surveillance was low in all hospitals, which potentially suggest overall awareness and guideline knowledge is lacking. For this specific population patient-tailored decision-making is primarily performed by oncologists and based on cardiotoxic treatment, cancer stage and comorbidities. Hospital-specific surveillance rates are, therefore, influenced by professionals’ decision-making and patients’ needs for complex care (e.g. less cardiac surveillance in patients with metastasized BC).

Although, a previous study of cardiac monitoring among BC patients suggests that most variance in cardiac surveillance is due to physician factors, as compared to patient factors [12], underlining the importance of clinicians’ knowledge and awareness of cardiac surveillance. In other medical fields where extensive research on medical variation is performed, diffused clinical protocols and subjective factors (e.g. preferences) appeared to be important causes of variation [22]. Objective clinical guidelines can increase physicians’ recognition of the importance of providing cardiac monitoring and reducing unwarranted hospital variation. Equality in care, regardless of the type of hospital patients are treated in, is imperative for improved health outcomes in BC patients and reduced healthcare costs. Additionally, a recent study found that oncologists’ views strongly differ from those of cardiologists’ as the former perceives cardiac surveillance solely as the management of symptomatic cancer therapy-related cardiac dysfunction and not as screening and monitoring of cardiotoxicity throughout the cancer treatment trajectory [23].

Cardiac surveillance is currently not well implemented in clinical practice although the importance of early cardiac damage detection has been established. In fact, CV mortality risk among BC survivors exceeds the mortality risk of the initial cancer or of recurrent disease [24, 25]. Detection and monitoring of cardiotoxicity in high-risk patients is pivotal to effectively manage negative cardiac effects and improve both prognosis and quality of life [6]. Recent studies have shown that early treatment with heart failure medication prevents decline in LV function, and this effect is lost when therapy is started at a later stadium [26]. In 2012, the European Society for Medical Oncology already emphasized the importance of cardiac monitoring both with echocardiography and cardiac biomarkers in high cardiotoxicity-risk patients [14]. Meanwhile, in 2016, the European Society of Cardiology (ESC) published a position paper providing a thorough review of the need for cardiac surveillance with biomarker testing and imaging to detect early cardiac damage [2]. However, these were not strict guidelines, and the tools to integrate this knowledge in clinical practice were lacking. For survivors of childhood malignancies, strict guidelines are implemented in clinical practice, and specialized cardiac surveillance centers have been established since the early 2000s [27]. Results indicate improved early diagnosis, prevention, and treatment of cardiac damage; improved health behavior and knowledge of patients; and lower rates of emergency department visits [28].

Regarding BC, there is evidence of incidence rates of cardiac damage, early detection (e.g. strain echocardiography, T1/T2 mapping CMR sequences, cardiac biomarkers), and treatment strategies [6]; however, this knowledge is not yet implemented in clinical protocols. To implement cardiac surveillance recommendations in current practice, awareness of its importance and multi-disciplinary collaboration are essential. We concur with Lancellotti et al. [29] that a comprehensive guideline is a major step forward to structure cardiac surveillance and improve equity in healthcare delivery between regions and hospitals, ultimately improving patients’ clinical outcomes.

### Strengths and limitations

A major strength of this study is the extensive nationwide claims database enabling us to explore HCU in detail with healthcare activity codes. The number of patients with newly diagnosed BC in our analysis corresponds with the number reported by the Dutch National Institute for Public Health and the Environment in 2013, which is approximately 16,900 [30]. A possible limitation is that our study is based on 2013–2015 data. The ESC position paper was published in 2016, and we were not able to evaluate whether this publication increased awareness and implementation of appropriate cardiac surveillance. However, since clinical protocols have not been adapted in the Netherlands, we do not expect that this position paper had a major impact on the clinical routine care of BC patients. One may argue that the lack of CV risk factors in the claims data is another limitation of the present study; however, according to current recommendations, cardiac surveillance should be provided to all patients receiving high-risk cardiotoxic treatment, independent of the presence of CV comorbidities or traditional CV risk factors [14]. The main focus of the present study is describing cardiac monitoring in newly diagnosed BC patients with a high CV risk based on the anti-cancer treatment they received and increasing awareness regarding the lack of implementation of routine cardiac monitoring. The presence of traditional CV risk factors should be used for further patient-tailored refinement of the risk stratification.

## Conclusion

Cardiac surveillance is not part of routine clinical care of BC patients in the Netherlands, resulting in low proportions of diagnostic procedures and a large variation of care delivery among hospitals. The overview of current practice provided by this study is a first step towards improving cardiac surveillance for women treated for BC. Cardiac surveillance, with baseline CV risk stratification, tailored monitoring, and treatment should be part of routine care for high-risk BC patients and should be incorporated in existing clinical guidelines or in new guidelines specifically aimed at monitoring cardiotoxicity.

## Supplementary information

**Additional file 1: Central illustration**. Cardiac surveillance during breast cancer treatment. * Percentage of patients for whom this type of care was registered at least once.

## Data Availability

The data that support the findings of this study are available from Statistics Netherlands but restrictions apply to the availability of these data, which were used under license for the current study, and so are not publicly available.

## Abbreviations

BC: Breast Cancer
CMR: Cardiovascular Magnetic Resonance
CT: Computed Tomography
CV: Cardiovascular
CVD: Cardiovascular Disease
ESC: European Society of Cardiology
ECG: Electrocardiogram
ESMO: European Society for Medical Oncology
GLS: Global Longitudinal Strain
HCU: Healthcare Utilization
LVEF: Left Ventricular Ejection Fraction
MUGA: Multigated Acquisition
STROBE: Strengthening the Reporting of Observational Studies in Epidemiology guideline
