# Supplementary figures and images for: Healthcare utilization and hospital variation in cardiac surveillance during breast cancer treatment: a nationwide prospective study in 5000 Dutch breast cancer patients
Source: Cardiooncology. 2020 Aug 8;6:14. doi: 10.1186/s40959-020-00068-6 (PMC7414746; doi:10.1186/s40959-020-00068-6)

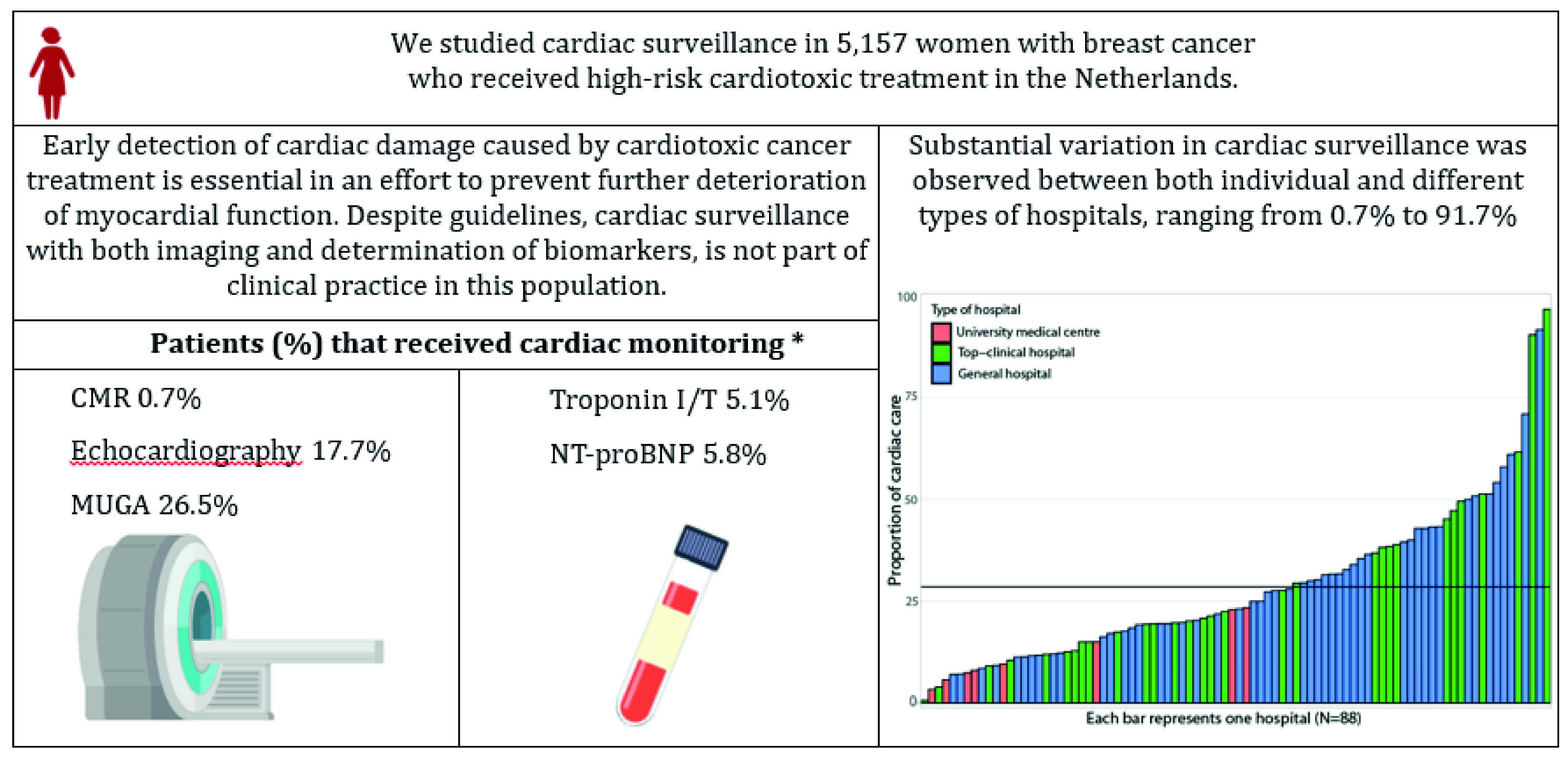

Supplement: Supplementary file 1 — Additional file 1: Central illustration. Cardiac surveillance during breast cancer treatment. * Percentage of patients for whom this type of care was registered at least once. [file 40959_2020_68_MOESM1_ESM.tif]
